# Cortical Structure in Pre-Readers at Cognitive Risk for Dyslexia: Baseline Differences and Response to Intervention

**DOI:** 10.1162/nol_a_00122

**Published:** 2024-06-03

**Authors:** Maria Economou, Femke Vanden Bempt, Shauni Van Herck, Toivo Glatz, Jan Wouters, Pol Ghesquière, Jolijn Vanderauwera, Maaike Vandermosten

**Affiliations:** Department of Neurosciences, KU Leuven, Leuven, Belgium

**Keywords:** cortical thickness, dyslexia, gray matter, intervention, plasticity, pre-readers

## Abstract

Early childhood is a critical period for structural brain development as well as an important window for the identification and remediation of reading difficulties. Recent research supports the implementation of interventions in at-risk populations as early as kindergarten or first grade, yet the neurocognitive mechanisms following such interventions remain understudied. To address this, we investigated cortical structure by means of anatomical MRI before and after a 12-week tablet-based intervention in: (1) at-risk children receiving phonics-based training (*n* = 29; *n* = 16 complete pre–post datasets), (2) at-risk children engaging with AC training (*n* = 24; *n* = 15 complete pre–post datasets) and (3) typically developing children (*n* = 25; *n* = 14 complete pre–post datasets) receiving no intervention. At baseline, we found higher surface area of the right supramarginal gyrus in at-risk children compared to typically developing peers, extending previous evidence that early anatomical differences exist in children who may later develop dyslexia. Our longitudinal analysis revealed significant post-intervention thickening of the left supramarginal gyrus, present exclusively in the intervention group but not the active control or typical control groups. Altogether, this study contributes new knowledge to our understanding of the brain morphology associated with cognitive risk for dyslexia and response to early intervention, which in turn raises new questions on how early anatomy and plasticity may shape the trajectories of long-term literacy development.

## INTRODUCTION

Literacy is a significant developmental milestone and an instrumental skill in modern society. Around 7% of school-aged children are diagnosed with developmental dyslexia, which means they face severe and persistent difficulties with reading and/or spelling, despite adequate instruction and learning opportunities (Peterson & Pennington, 2015). Behavioral research indicates that phonics-based interventions can be beneficial for individuals with dyslexia (Galuschka et al., 2014; Snowling & Hulme, 2011) and even more so if taking place in early stages of reading development such as kindergarten and first grade (Lovett et al., 2017; Wanzek & Vaughn, 2007). In the past two decades, great efforts have been made to characterize the neuroanatomical profile of dyslexia with a few studies focusing on learning-related neuroplasticity in struggling readers following explicit intervention (for a review see Perdue et al., 2022). Yet, a gap in knowledge remains concerning the impact of preventive intervention, that is, intervention that takes place before the onset of formal reading instruction. Here, we (1) assessed anatomical differences in 5-year-old preliterate children with and without an increased cognitive risk of developing dyslexia and (2) examined whether a preventive intervention program during kindergarten is associated with cortical plasticity in at-risk children.

Several neuroimaging studies have investigated the gray matter correlates of reading, revealing widespread findings. In adults, the literature points to mostly positive associations between reading-related skills and gray matter structure in bilateral perisylvian and left occipito-temporal regions (He et al., 2013; Johns et al., 2018; Pernet et al., 2009). In children, a large-scale cross-linguistic study reported a negative correlation between reading accuracy and gray matter volume of the left cerebellum, yet at the same time a positive correlation with volume of the left SMG in typical, but not dyslexic readers (Jednoróg et al., 2015). In contrast to previously reported findings, a large-scale study in English-speaking children and adolescents found no associations between gray matter volume and single word reading ability (Torre & Eden, 2019). However, when subdividing the sample in age- and sex-based groups, the authors found sex-specific results in the older participants (aged 15–22 years) showing that reading was positively correlated to gray matter volume in the left fusiform for females and in the right superior temporal gyrus for males (Torre & Eden, 2019). More recently, studies with children and adolescents have reported positive associations between reading scores and cortical thickness in the left SMG and postcentral gyrus, while positive associations were also reported bilaterally for inferior temporal gyrus, superior temporal gyrus and occipito-temporal regions including the fusiform gyrus (Perdue et al., 2020; Williams et al., 2018; Xia et al., 2018). Interestingly, in Xia et al. (2018) specific subcomponents of reading such as phonetic representation, phonological awareness and orthography–phonology mapping were uniquely predicted by cortical thickness in the left inferior, middle and superior temporal gyri, respectively). In sum, the pediatric literature points toward primarily positive associations between reading and cortical structure bilaterally, particularly implicating occipito-temporal and perisylvian cortex, but negative associations and null results have also been reported.

Next to associations between cortical anatomy and reading-related skills, previous research has demonstrated that individuals with dyslexia show atypical gray matter structure at the group level, when compared to typically reading individuals. Concerning gray matter volume, studies in adults have shown that dyslexia was associated with volume reductions in the left basal ganglia and bilaterally in the planum temporale, cerebellum and inferior, middle and superior temporal gyri (Brambati et al., 2004; Kujala et al., 2021; Silani et al., 2005; Steinbrink et al., 2008; Vinckenbosch et al., 2005). At the same time, higher volume in individuals with dyslexia compared to controls was found in bilateral precentral gyri (Vinckenbosch et al., 2005). In children and adolescents, dyslexia-related gray matter volume reductions were reported bilaterally in the cerebellum, fusiform gyri, supramarginal gyri, superior temporal gyri and inferior frontal gyri, as well as the right precentral gyrus, left middle temporal gyrus and left superior occipital gyrus (Eckert et al., 2003; Krafnick et al., 2014; Kronbichler & Kronbichler, 2018; Xia et al., 2016). Meta-analyses based on the voxel-based morphometry literature have identified convergence among studies mainly in bilateral temporo-parietal, left occipito-temporal and left orbitofrontal regions (Eckert et al., 2016; Linkersdörfer et al., 2012; Richlan et al., 2013).

Alongside volume, other cortical features have also been investigated in children and adults with dyslexia. Here, studies have identified lower cortical thickness in dyslexia relative to typically reading individuals in the left insula (Kujala et al., 2021), bilateral occipito-parietal cortex, and right orbitofrontal, paracentral and inferior temporal regions (Williams et al., 2018). Increased cortical thickness in the dyslexia group has also been reported in relation to a typical control (TC) group, specifically in the left fusiform gyrus and bilateral supramarginal gyri (Partanen et al., 2020). Although less frequently investigated, gyrification differences have also been reported in children with dyslexia, pointing toward increased gyrification relative to controls in left inferior parietal, lateral occipital and inferior temporal cortices, as well as right superior frontal cortex (Williams et al., 2018). A few studies have also examined group variation in cortical surface area, revealing differences in the asymmetry of the planum temporale surface area (Altarelli et al., 2014) and the right fusiform gyrus (Partanen et al., 2020). To summarize, studies have identified bilateral structural gray matter differences between individuals with and without dyslexia, which implicate the auditory cortex, temporo-parietal and occipito-temporal regions, and less consistently frontal, occipito-parietal, subcortical regions and the cerebellum. We note that inconsistencies in the literature have also been described, which can be, at least partly, attributed to methodological (e.g., sample- or analysis-related) and cross-linguistic differences (Moreau et al., 2019; Ramus et al., 2018). More specifically, the sample size and age range of participants, whether studies have used voxel-based morphometry or surface-based models and whole-brain or region-of-interest analysis, how dyslexia classification and binary grouping is defined in each study, and whether the control groups include age-matched and/or reading level-matched individuals are all sources of inconsistency across dyslexia studies examining group-level gray matter differences. Although there is variability on the precise location and direction of the reported differences, the pattern of results is generally consistent with what is broadly described as the anatomical reading network (Chyl et al., 2021; Jobard et al., 2003; Wandell & Yeatman, 2013).

Importantly, there is growing evidence across several independent samples suggesting that atypical gray matter structure may be present even before the start of reading instruction, and thus contradicting the idea that the group-level neuroanatomical differences observed in dyslexia are solely a consequence of diminished reading experience and exposure. At the pre-reading stage for instance, familial risk for dyslexia has been associated with smaller inferior and middle temporal gyri bilaterally (Beelen et al., 2019), reduced cortical thickness in the left SMG and left occipito-temporal cortex (Kraft et al., 2015), atypical asymmetry of the planum temporale (Vanderauwera et al., 2018), as well as atypical sulcal patterns (Im et al., 2016). In addition, family history of dyslexia has been linked to reduced gray matter volume in pre-readers in regions spanning bilateral prefrontal regions, the left fusiform gyrus, right lingual gyrus, and left occipito-temporal and bilateral temporo-parietal areas (Black et al., 2012; Raschle et al., 2011).

More direct evidence for early dyslexia-related anatomical differences is provided by studies in which children are followed up for reading, allowing a dyslexia classification. Two of these studies have shown structural differences in the auditory cortex between pre-readers who later developed dyslexia and pre-readers who later developed typical reading skills (Clark et al., 2014; Kuhl et al., 2020), though the findings of Clark and colleagues should be interpreted with caution due to the very small sample size. In addition, Beelen et al. (2019) found that pre-readers who later develop dyslexia have bilaterally smaller fusiform gyri, with no differences in the gray matter trajectories of these regions being observed throughout primary school (Phan et al., 2021), suggesting that the original deficits in the left reading network remain present throughout primary school.

Can these early putative gray matter alterations become targets for intervention? Given the demonstrated efficacy of literacy intervention for remediation of reading difficulties (Galuschka et al., 2014), a preventive training might offer the potential to alter the neurodevelopmental trajectories of (a)typical reading. To date, very few studies have directly investigated gray matter changes specifically linked to reading intervention in children. Krafnick et al. (2011) examined changes in gray matter volume following an intensive reading intervention in 11 children with dyslexia (ages 7–11). Increased volume was observed in several clusters in the brain from pre- to post-intervention, but not during an 8-week waiting control period. These changes were located specifically in the left anterior fusiform gyrus extending into the hippocampus, left precuneus, right hippocampus and right anterior cerebellum. The intervention-induced changes reported by Krafnick et al. (2011) are consistent with the role of the left ventral occipito-temporal (vOT) cortex in word-specific processing and notably extend further into noncanonical reading regions, which the authors speculate reflect general learning mechanisms and the memory retrieval or visual imagery aspects of the specific intervention program that was followed. This study constitutes the first indication of gray matter plasticity after successful reading intervention; however, replication of these findings is necessary given the comparatively small sample size and the absence of any control group. More recently, Romeo et al. (2018) examined changes in cortical thickness in children with reading disability (aged 6–9 years) receiving a 6-week reading intervention. The authors reported no overall differences in thickness changes between the intervention and waiting control groups; however, within the intervention group and specifically between treatment responders and nonresponders, differences were observed. Responders showed significantly larger cortical thickening than nonresponders in several bilateral clusters spanning middle/inferior temporal cortex, supramarginal/angular regions, precentral and paracentral regions, as well as the right superior temporal gyrus. On the other hand, cortical thickness did not change significantly in nonresponders and controls. In contrast to the studies by Krafnick et al. (2014) and Romeo et al. (2018), Partanen et al. (2020) investigated both thickness and volume measures and found no evidence of gray matter plasticity following a 3-month reading intervention in school-aged children with dyslexia compared to typical readers. Hence, while these studies provide some evidence of changes in gray matter structure in response to intervention, diverse (and null) effects have also been reported, suggesting little convergence on the magnitude and location of the intervention-related changes. Importantly, there is a gap in knowledge concerning neuroplasticity in response to intervention that occurs in the earliest stages of reading development.

The present study aims to address this gap by reporting on an early literacy intervention study in 5-year-old pre-reading children with an increased cognitive risk for developing dyslexia. First, we examined whether anatomical differences in cortical measures exist at baseline, between at-risk and typically developing children. Second, by means of a longitudinal design, we investigated whether a 12-week kindergarten intervention is associated with cortical plasticity in at-risk children receiving early literacy training, relative to at-risk children engaging with non-literacy training and typically developing children. Our analysis focused on a set of regions of interest (ROIs) selected a priori, that emerged in the literature as structurally and functionally relevant areas supporting associations with (early) literacy skills and intervention-related changes. These included: bilateral fusiform gyrus; inferior, middle and superior temporal gyri; SMG; and inferior frontal gyrus (pars opercularis and pars triangularis). Further, we decided to characterize gray matter structure by zooming into the two individual subcomponents of gray matter volume separately, that is, cortical surface area (SA) and cortical thickness (CT). Although both measures change across the lifespan (Tamnes et al., 2017), they seem to be differentially influenced by prenatal factors and various environmental or demographic variables (Jha et al., 2019; Raznahan et al., 2012). For instance, drastic surface area changes are observed during late fetal development as a result of cortical folding (Kapellou et al., 2006), whereas cortical thickness undergoes dynamic changes as a function of age, which seems to occur in concert with cognitive development (Frangou et al., 2021; Shaw et al., 2006; Sowell et al., 2004). These findings, coupled with evidence of distinct genetic determinants of cortical thickness and surface area (Panizzon et al., 2009) and more prenatal than later environmental influences on surface area development (Jha et al., 2019), lead us to hypothesize that if learning-related changes are observed, these should be expected in cortical thickness rather than surface area.

## MATERIALS AND METHODS

The data reported here were collected as part of a larger longitudinal intervention project investigating the impact of early literacy training on behavioral and neural outcomes. Findings from an overlapping sample who participated in the intervention study were reported in previous publications (Economou et al., 2022; Vanden Bempt et al., 2021). Behaviorally, the training led to specific improvements in receptive and productive letter sound knowledge and word decoding, beyond typical maturation (Vanden Bempt et al., 2021). The current study focuses on examining risk- and intervention-related differences in cortical gray matter, assessed by longitudinal T1-weighted magnetic resonance imaging (MRI) measurements.

### Participants

The participants of this study were 90 monolingual Flemish Dutch-speaking children recruited as part of a broader intervention study (Economou et al., 2022). All children were in their third year of kindergarten, which is the last year before entering primary school and therefore before receiving formal reading instruction in accordance with Flemish legislation. Inclusion criteria required participants to have no prior known hearing or neurological problems, no formal diagnosis or risk for ADHD (Smidts & Oosterlaan, 2007) and no history of speech and language therapy. In addition, all children had received comparable preschool education by the time of recruitment. The data reported in this study were collected in two waves between December 2018 and June 2019, therefore before the start of the COVID-19 pandemic.

The sample consisted of 60 children with and 30 children without an increased cognitive risk for dyslexia. Risk status was determined in a first screening session in which letter knowledge, phonological awareness and rapid automatized naming were assessed, as relevant predictors of early reading (Caravolas et al., 2012; Puolakanaho et al., 2007) and is described in more detail in previous publications (Vanden Bempt et al., 2021; Van Herck et al., 2021; Verwimp et al., 2020). Briefly, percentile scores for the early literacy measures were calculated in a sample of 1,091 Flemish kindergarteners. Children were classified as at-risk if they performed below the 30th percentile on two out of the three measures and below the 40th percentile on letter knowledge. They were classified as not having a risk if they performed above the 40th percentile on all measures. The screening percentile scores of the current sample are reported in the Results section (see Table 1).

**Figure 1.**
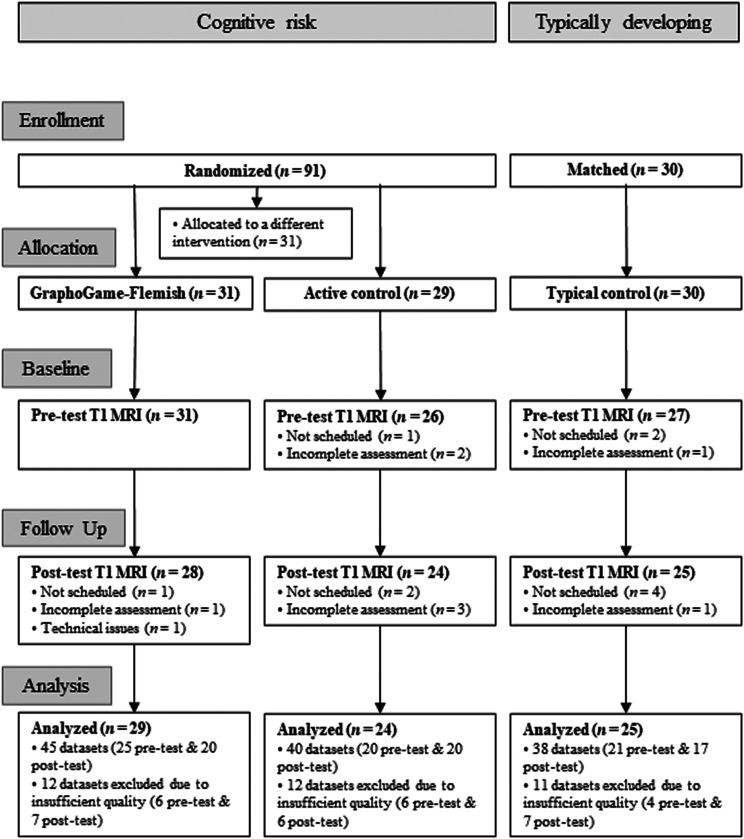
Adapted Consolidated Standards of Reporting Trials (CONSORT) diagram summarizing the flow of participants in the study.

Of the 60 at-risk children, 31 were randomly assigned to a tablet-based intervention that aimed at training early literacy skills, while the other 29 children engaged with a non-literacy control intervention (see below). A block randomization procedure was used to assign at-risk children to intervention conditions, while balancing the factors of sex, birth trimester and school across groups. The group of typically developing children (*n* = 30) was matched for sex, age and nonverbal intelligence (as measured by the Raven’s colored progressive matrices test; Raven et al., 1984) and participated in the behavioral and MRI assessments but did not receive any intervention. A detailed description of participant flow in the study is provided in Figure 1. For all cognitive and neuroimaging assessments, written informed consent and verbal assent were obtained from all primary caregivers and children, respectively. This study was approved by the Medical Ethics Committee of the university hospital of KU Leuven.

### Intervention

The tablet-based early literacy and control interventions have been described in detail elsewhere (Economou et al., 2022; Glatz et al., 2022). For the specific purposes of our study the GraphoGame interface (Richardson & Lyytinen, 2014) was adapted to create a new tablet-based version (GraphoGame-Flemish; GG-FL), which is appropriate for Flemish Dutch-speaking children without any formal reading instruction. In brief, the game content consisted of grapheme and phoneme introduction, exercises of visual discrimination of graphemes and auditory discrimination of phonemes, gradually building up to exercises of phoneme blending, phoneme counting, grapheme–phoneme coupling and early reading and spelling. Children in the active control (AC) group engaged instead with commercially available games which were age-appropriate and did not train early literacy skills. Specifically, they were able to choose from one of six games from brands such as LEGO and Playmobil during the training session.

All training took place at home where participating children and their families were instructed to play for 15 minutes a day, six days a week for a total duration of 12 weeks. As part of the larger longitudinal project, both intervention groups received an additional story listening game, which they were asked to use for 10 minutes a day in addition to their normal training schedule (Vanden Bempt et al., 2022). The effects of story listening are not the focus of the current study and will not be discussed further. Note that the two groups did not differ in the amount of story listening or the total amount of intervention-related tablet exposure (Vanden Bempt et al., 2022). For all games, information regarding the start and end of gaming sessions was logged, which allowed the extraction of variables such as the duration and frequency of training sessions. These log files were transmitted daily to a central university server. The two intervention groups had comparable exposure of their respective games over the training period and did not differ in the amount of story listening or tablet use (Economou et al., 2022; Vanden Bempt et al., 2021). Contact with the parents was maintained throughout the intervention in the case of technical or motivational/compliance issues.

### MRI Data Acquisition

Participants (*n* = 90) were invited for two MRI sessions, one before and one shortly after the training phase. All images were acquired on a 3T Philips Achieva MRI scanner (Philips, Best, The Netherlands) using a 32-channel head coil and identical acquisition parameters. Anatomical 3D T1-weighted images were acquired using a CS-SENSE TFE (compressed sensing-sensitivity encoding turbo field echo) sequence with following parameters: 240 sagittal slices, isotropic voxel size 0.9 mm^3^, repetition time/echo time (ms) = 9.1/4.2, acquisition time = 3 min 30 s. An age-appropriate preparation protocol was used to familiarize the children with the scanning procedure and minimize motion artifacts.

At pre-test, T1-weighted images were not available for six individuals who either did not schedule (*n* = 3) or did not complete (*n* = 3) their MRI assessments. At post-test, T1-weighted images were not available for seven participants who did not schedule a second visit, five participants who did not complete the MRI assessment and one dataset that was compromised due to technical issues (see Figure 1). Visual inspection of the T1-weighted images revealed that 35 datasets (17 at pre-test and 18 at post-test) showed severe motion as defined by the criteria described in Blumenthal et al. (2002), that is, severe ringing and blurring artifacts, due to which they were deemed unusable for subsequent analysis. Quality control inspection was done twice by the first author with a gap of two years between the ratings. Note that the rater had less than two years of experience at the time of the first rating. An absolute intra-rater agreement of 0.87 (95% CI [0.80, 0.92]) was reached. A subset of images with moderate to severe quality artifacts with disagreement in intra-rater ratings (*n* = 12) was additionally inspected by a second, independent rater with similar background, in order to reach a final decision. Besides quality issues, three datasets had to be discarded due to preprocessing failure. Thus, the present study reports analyzed data from 78 subjects (total *N* = 123 datasets). Note that not all participants included in the analyses had complete pre–post usable datasets. Concretely, complete pre–post datasets were available for *n* = 16 in the GG-FL group, *n* = 15 in the AC group and *n* = 14 in the TC group. Nevertheless, all available data were used for further analyses following quality control, given that our choice of statistical framework is compatible with missing observations. The flowchart shown in Figure 1 provides a detailed breakdown of the data used in analysis per group and time point.

### MRI Data Processing

Images were processed using the FreeSurfer 6.0 image analysis suite (Fischl, 2012), which is documented and freely available online at https://surfer.nmr.mgh.harvard.edu/. The technical details of these procedures were described in detail in prior publications (Dale et al., 1999; Fischl et al., 1999; Fischl et al., 2002). Briefly, the processing stream includes motion correction (Reuter et al., 2010), removal of non-brain tissue using a hybrid watershed/surface deformation procedure (Ségonne et al., 2004), automated Talairach transformation, nonparametric nonuniform intensity normalization (Sled et al., 1998), tessellation of the gray/white matter boundary, automated topology correction (Fischl et al., 2001; Ségonne et al., 2007) and surface deformation following intensity gradients to optimally place the gray/white and gray/cerebrospinal fluid borders at the location where the greatest shift in intensity defines the transition to the other tissue class (Dale et al., 1999; Dale & Sereno, 1993; Fischl & Dale, 2000). Each cortical model was registered to a spherical atlas using individual cortical folding patterns to match cortical geometry across participants (Dale et al., 1999).

Images were then processed using FreeSurfer 6.0’s longitudinal stream, which has been shown to be more sensitive than the cross-sectional pipeline in detecting cortical changes in longitudinal designs (Reuter et al., 2012) and is routinely used in developmental studies (Mills et al., 2016; Romeo et al., 2018; Tamnes et al., 2017; Wierenga et al., 2014). During this process, an unbiased within-subject template space and image is created using robust, inverse consistent registration (Reuter et al., 2010). Several processing steps, such as skull stripping, Talairach transforms, atlas registration as well as spherical surface maps and parcellations were then initialized with common information from the within-subject template, significantly increasing reliability and statistical power (Reuter et al., 2012). Data from participants with only one available time point were also processed using the longitudinal stream to ensure that all images included in statistical analysis undergo the same processing steps (Bernal-Rusiel et al., 2013). To allow for a quantitative assessment of image quality, a measure of signal-to-noise ratio (SNR) was derived using FreeSurfer tools, calculated based on the white matter segmentation.

From the surface-based cortical representations, measurements for cortical thickness and surface area were extracted for further statistical analysis. CT was quantified as the distance between the white surface (white and gray matter boundary) and the pial surface (gray matter and outer cerebrospinal fluid/dura boundary) of the cortex, while SA was quantified as the size of the white surface (Fischl & Dale, 2000; Fischl et al., 1999). Measures of CT and SA derived from the FreeSurfer surface-based processing stream have been validated and shown good test–retest reliability across multiple sites, scanner manufacturers and field strengths (Haddad et al., 2023; Han et al., 2006; Knussmann et al., 2022; Reuter et al., 2012).

Regional CT and SA measurements were obtained from ROIs selected as part of FreeSurfer’s automatic parcellation using the Desikan-Killiany atlas (Desikan et al., 2006). Given the aim of this study to examine changes in brain structure in response to receiving early literacy (i.e., GG-FL) training, ROIs were predefined based on their functional relevance in emerging literacy. More specifically, we focused on a set of regions that are part of the reading circuitry, identified as important during the initial specialization of the visual cortex to process print-specific stimuli, as well as the integration of audiovisual information needed for successful learning and automation of grapheme–phoneme correspondences. In line with findings from independent research groups, this led to the selection of: temporal cortex (inferior, middle and superior temporal gyri), occipito-temporal cortex (fusiform gyrus), inferior parietal cortex (SMG) and inferior frontal cortex (pars opercularis and pars triangularis) (Beelen et al., 2019; Brem et al., 2010; Chyl et al., 2021; Dehaene et al., 2010; Karipidis et al., 2017; Karipidis et al., 2021; Yamada et al., 2011). Given the evidence of bilateral engagement in early literacy (for a concise review see Richlan, 2019), as well as the evidence for bilateral literacy intervention-induced changes in gray matter structure (Krafnick et al., 2011; Romeo et al., 2018), both left- and right-hemispheric regions were investigated.

### Statistical Analysis

All reported statistical analyses were performed in R version 4.0.0 (R Core Team, 2020), with the alpha level set at 0.05. First, risk-related differences in cortical measures were examined before the start of the intervention. For the purpose of this baseline analysis, the at-risk intervention group (GG-FL) and at-risk AC group were combined (*n* = 45) and compared to the TC group (*n* = 21). Regional measures of CT and SA were compared between at-risk and typically developing children per ROI using linear models. There was no a priori trimming of the data, but if visual inspection revealed violated model assumptions regarding properties of residuals after fitting, observations leading to residuals more than 2 *SD* above or below the prediction were removed (this amounted to no more than four datasets) and the model was refitted.

To assess the effects of training on cortical measures, linear mixed-effects models (LMMs) were built for each ROI with the independent variables of session (pre-test, post-test), group (GG-FL, AC, TC) and the interaction between them. By-subject random intercepts were included to account for within-subject variability. In case of issues with model diagnostics such as non-normal and nonsymmetric residual distribution, robust LMMs were applied (Koller, 2016). Separate models were constructed for each structural measure (CT, SA). All LMMs were fitted using the function lmer from the R package *lme4* (Bates et al., 2015).

In all models, statistical inference was conducted by calculating 95% confidence intervals and *p* values using parametric bootstrapping (1,000 iterations) as implemented in the R package *parameters*. Where applicable (i.e., in the presence of significant group-by-session interactions), a follow-up analysis was conducted on pairwise comparisons of the estimated marginal means (R package *emmeans*), applying false discovery rate (FDR) correction (*q* = 0.05). Standardized regression coefficients (*β*) are reported for all models, which enables their interpretation as Cohen’s *d* effect sizes. In line with prior literature (see Altarelli et al., 2014), sex was added as an independent variable in both baseline as well as longitudinal analyses. In addition, in all models where regional SA as dependent variable, total hemispheric SA was included as an independent variable. Age was not further statistically accounted for given that our study design includes a limited age range (all children were born in the same year). Lastly, linear models were fitted to compare groups on measures that could influence the interpretation of potential risk- or intervention-related effects, including global cortical measures (average CT and total SA) and quantitative image quality metrics (SNR). Given a potential session effect on image quality (e.g., due to moving less in the post-test session), we additionally examined the effect of session on SNR using a LMM analysis.

## RESULTS

Participant demographic and training characteristics are reported in Table 1. The three groups did not differ significantly with respect to sex, nonverbal intelligence, handedness, age at either MRI timepoint, socio-economic status (indexed via maternal education level) or parent-reported home literacy environment variables. Although more children in the GG-FL group had a familial risk for dyslexia compared to the other groups, this was not further accounted for given the very low occurrence in our sample (*n* = 6). As shown in Table 1, both at-risk groups (GG-FL, AC) had overall comparable game exposure and total training duration and the majority of children completed at least 90% of the intended intervention amount (estimated at 18 hr). The median training duration was in line with the intended duration of the program (12 wk), though the range indicates variability among children. With respect to image quality, no differences were observed in Freesurfer-derived SNR between at-risk and typically developing children at baseline (*β* = −0.29, 95% CI [−0.85, 0.23], *p* = 0.260). The LMM analysis of SNR indicated no evidence for an effect of group (*F*(2, 73) = 0.23, *p* = 0.80), session (*F*(1, 54) = 2.59, *p* = 0.11) or group-by-session interaction (*F*(2, 54) = 1.61, *p* = 0.21) on SNR. Here we report results from the longitudinal FreeSurfer output, but the results were almost identical when the analyses were performed with the cross-sectional stream output.

**Table 1.** Participant demographic and training characteristics.

Variable	Group			*p* value^b^
	GG-Flemish (*n* = 29)^a^	Active control (*n* = 25)^a^	Typical control (*n* = 24)^a^	
Sex (female/male)	14/15	11/14	14/10	0.588
Familial risk	5	1	0	0.046
Nonverbal intelligence	102 (78–133)	96 (79–126)	102 (78–133)	0.528
Handedness^c^				0.964
Left/right/ambidextrous	2/26/1	2/21/2	2/20/2	
Age at pre-test MRI (months)	64 (60–71)	65 (60–72)	66 (62–73)	0.109
Age at post-test MRI (months)	69 (65–76)	69 (65–76)	70 (65–76)	0.849
Socioeconomic status^d^				0.099
Low/middle/high/unknown	7/11/11/0	8/12/4/1	3/8/13/0	
Letter knowledge (percentile screening score)	23 (11–36)	21 (11–29)	80 (40–100)	
Phonological awareness (percentile screening score)	27 (8–71)	21 (4–67)	84 (44–100)	
Rapid automatized naming (percentile screening score)	23 (3–92)	27 (4–84)	64 (40–99)	
Duration of joint literate activities^e^	−0.8 (−0.9–1.3)	−0.7 (−0.9–1.3)	−0.7 (−0.9–1.3)	0.572
Frequency of joint literate activities^e^	−0.1 (−2.1–1.6)	−0.1 (−2.1–1.2)	0.2 (−2.1–1.5)	0.266
Duration and frequency of reading alone^e^	−0.5 (−0.8–2.0)	−0.5 (−0.8–1.9)	−0.4 (−0.8–1.8)	0.753
Number of books at home^e^	0.7 (−2.2–1.8)	0.1 (−2.2–1.7)	0.0 (−2.1–1.8)	0.130
Baseline left mean cortical thickness (mm)	2.99 (2.70–3.14)	3.03 (2.85–3.20)	2.95 (2.79–3.08)	0.252
Baseline right mean cortical thickness (mm)	2.98 (2.72–3.19)	2.99 (2.85–3.19)	2.98 (2.75–3.11)	0.714
Baseline left total surface area (mm^3^)	79 (70–94)	81 (67–101)	83 (74–99)	0.510
Baseline right total surface area (mm^3^)	80 (70–94)	81 (68–101)	83 (73–98)	0.396
Training exposure (hours)	16 (3–25)	18 (6–28)	–	0.319
Training period (weeks)	12 (6–19)	13 (10–20)	–	0.371
Proportion of completed intervention (%)^f^	91 (15–137)	101 (32–158)	–	0.319

^a^ Counts (*n*) or median (range).

^b^ Group differences were assessed using a Kruskal-Wallis rank sum test for age, nonverbal intelligence, global cortical measures, home literacy variables and training characteristics, a Pearson’s Chi-squared test for sex, handedness and socioeconomic status, and a Fisher’s exact test for familial risk.

^c^ Based on a subset of questions adapted from the Edinburgh Handedness Inventory (Oldfield, 1971).

^d^ Based on maternal educational level (low = no extra degree after secondary school; middle = professional bachelor/academic bachelor; high = Master or PhD).

^e^ Standardized factor scores based on parental screening questionnaires.

^f^ The proportion of completed intervention is calculated as the number of hours spent on-task divided by the total amount of hours children were instructed to play during 12 weeks (estimated at 18 hr).

### Cognitive Risk-Related Effects Before the Intervention

**Figure 2.**
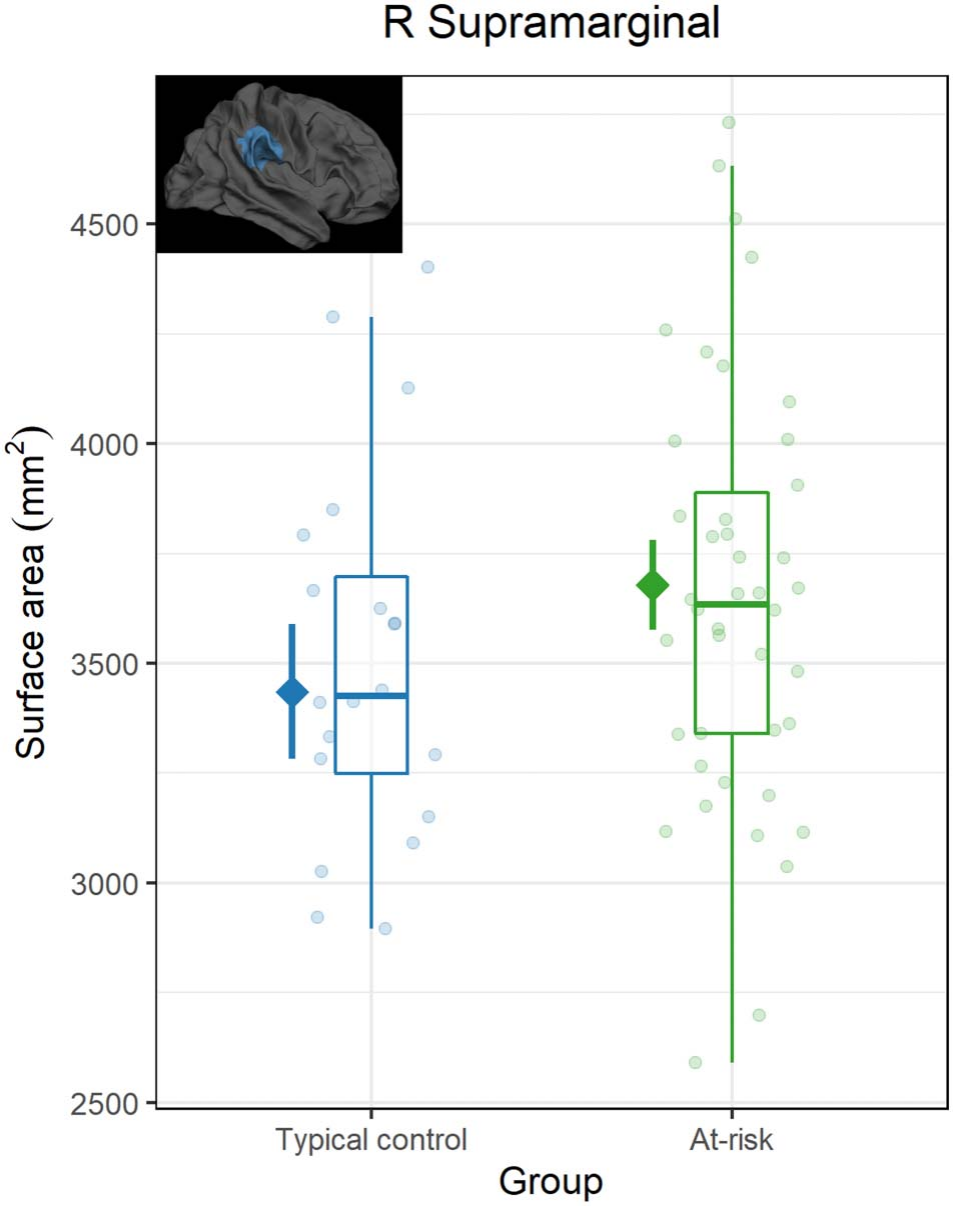
Surface area of the right supramarginal gyrus at baseline. Boxplots and semitransparent dots represent raw data, while solid diamonds and lines represent the model-based trimmed estimated marginal means for each group (cognitive risk and typical control) along with a 95% confidence interval.

First, baseline global cortical measures were compared between the at-risk and TC group. No average CT differences were observed in the left (*β* = 0.31, 95% CI [−0.24, 0.78], *p* = 0.242) or the right (*β* = −0.21, 95% CI [−0.71, 0.23], *p* = 0.352) hemisphere. Similar results were observed in total SA comparisons, with no differences found in either the left (*β* = −0.21, 95% CI [−0.71, 0.23], *p* = 0.352) or the right (*β* = −0.2, 95% CI [−0.67, 0.28], *p* = 0.438) hemisphere. In a second step, baseline regional group differences between at-risk and typically developing children were examined, revealing no evidence of cognitive risk effects on CT in the investigated ROIs. An effect of risk on SA was observed in the right SMG (*β* = 0.52, 95% CI [0.13, 0.93], *p*_boot_ = 0.006), revealing higher SA in at-risk children compared to typically developing children (Figure 2). No other group differences were observed in SA.

### Intervention Effects on Cortical Measures

#### Cortical thickness

**Figure 3.**
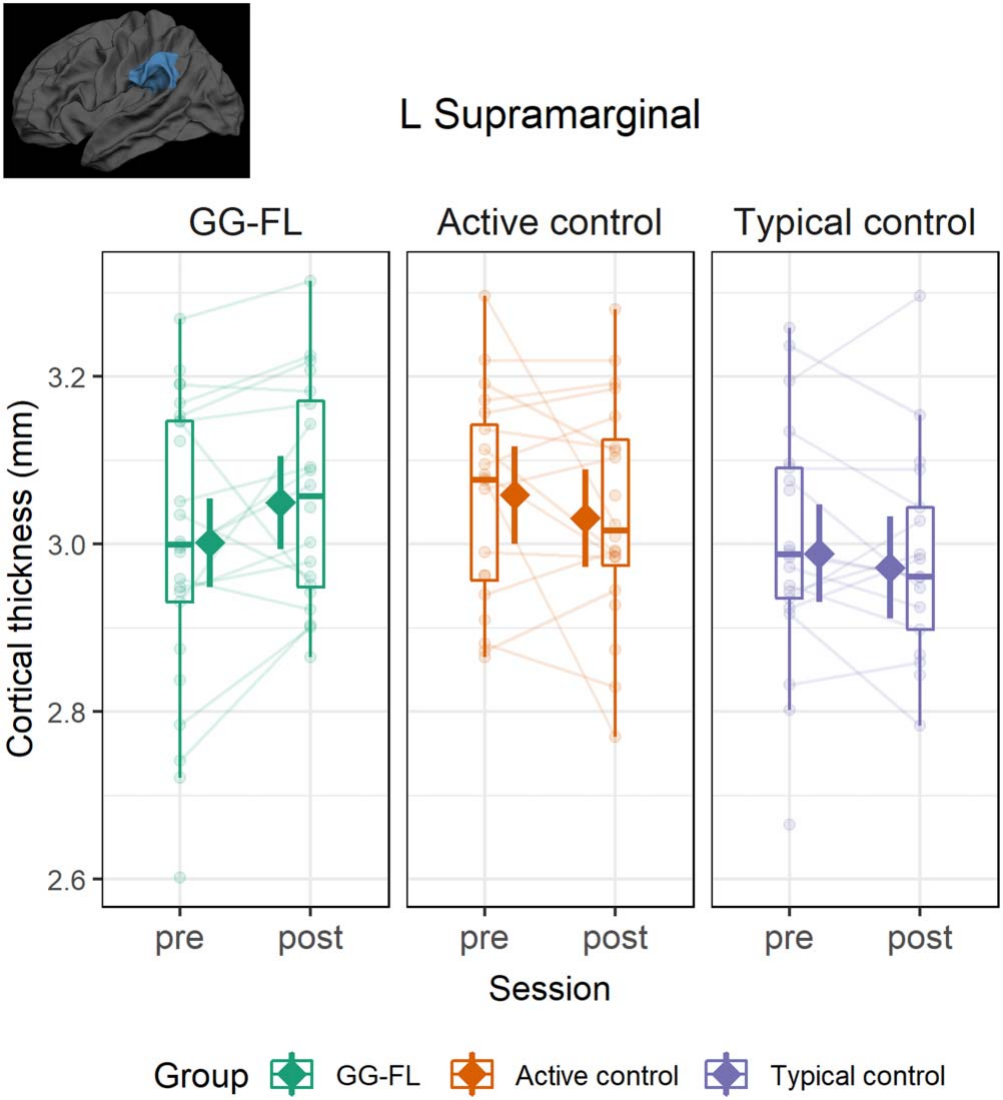
GraphoGame-Flemish (GG-FL) training-specific increase in cortical thickness of the left supramarginal gyrus following intervention. Boxplots and semitransparent dots and lines represent individual subject longitudinal changes. The solid diamonds represent model-predicted thickness values with 95% confidence bands across the two time points for the three groups.

Longitudinal changes in CT and SA from pre- to post-test were compared in the three groups (GG-FL, AC, TC) by means of linear mixed-effects models. As shown in Figure 3, we found evidence of a group-by-session interaction effect for CT of the left SMG (*β* = 0.20, 95% CI [0.04, 0.36], *t*(116) = 2.47, *p* = 0.014), showing that increased thickness was found over time in the GG-FL group (ΔCT = 48 *μ*m, *SE* = 0.02, 95% asymptotic CI [0.01, 0.09], *z* = 2.26, *p* = 0.024, *p*_FDR_ = 0.071), but not in the other two groups (AC: ΔCT = −28 *μ*m, 95% asymptotic CI [−0.07, 0.02], *z* = −1.25, *p* = 0.211, *p*_FDR_ = 0.316; TC: ΔCT = −17 *μ*m, 95% asymptotic CI [−0.06, 0.03], *z* = −0.73, *p* = 0.463, *p*_FDR_ = 0.463).

**Figure 4.**
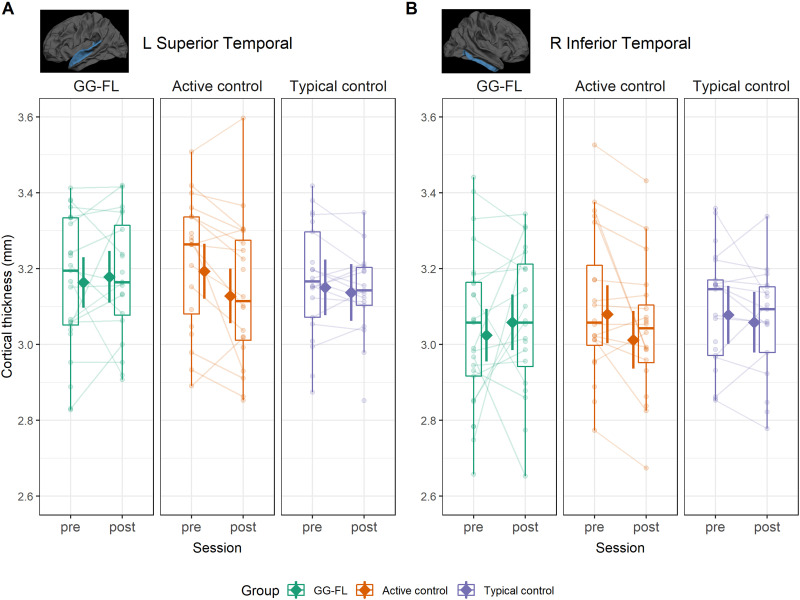
Active control specific group differences in longitudinal cortical thickness change following intervention. Boxplots and semitransparent dots and lines represent individual subject longitudinal changes. The solid diamonds represent model-predicted thickness values with 95% confidence bands across the two time points for the three groups. (A) Results for the left (L) superior temporal gyrus. (B) Results for the right (R) inferior temporal gyrus.

Group-by-session interaction effects were also observed in the left superior temporal (*β* = 0.18, 95% CI [0.05, 0.31], *t*(116) = 2.76, *p* = 0.006) and right inferior temporal (*β* = 0.21, 95% CI [0.03, 0.39], *t*(116) = 2.3, *p* = 0.021) regions. These results are visualized in Figure 4. Follow-up analyses indicate that in the left superior temporal region, there was decreased thickness in the AC group (ΔCT = −65 *μ*m, 95% asymptotic CI [−0.11, −0.02], *z* = −3.14, *p* = 0.002, *p*_FDR_ = 0.005), which was not observed in the GG-FL group (ΔCT = 15 *μ*m, 95% asymptotic CI [−0.02, 0.05], *z* = 0.73, , *p* = 0.466, *p*_FDR_ = 0.546) or the TC group (ΔCT = −13 *μ*m, 95% asymptotic CI [−0.06, 0.03], *z* = −0.6, *p* = 0.546, *p*_FDR_ = 0.546). Similarly, in the right inferior temporal region, the AC group exhibited thinning over time (ΔCT = −68 *μ*m, 95% asymptotic CI [−0.13, −0.01], *z* = −2.12, *p* = 0.034, *p*_FDR_ = 0.101), in contrast to the GG-FL group (ΔCT = 34 *μ*m, 95% asymptotic CI [−0.03, 0.09], *z* = 1.12, *p* = 0.265, *p*_FDR_ = 0.397) and the TC group (ΔCT = −19 *μ*m, 95% asymptotic CI [−0.08, 0.05], *z* = −0.59, *p* = 0.558, *p*_FDR_ = 0.558). No other training-related effects were observed, as indicated by the absence of any group-by-session interactions.

#### Surface area

**Figure 5.**
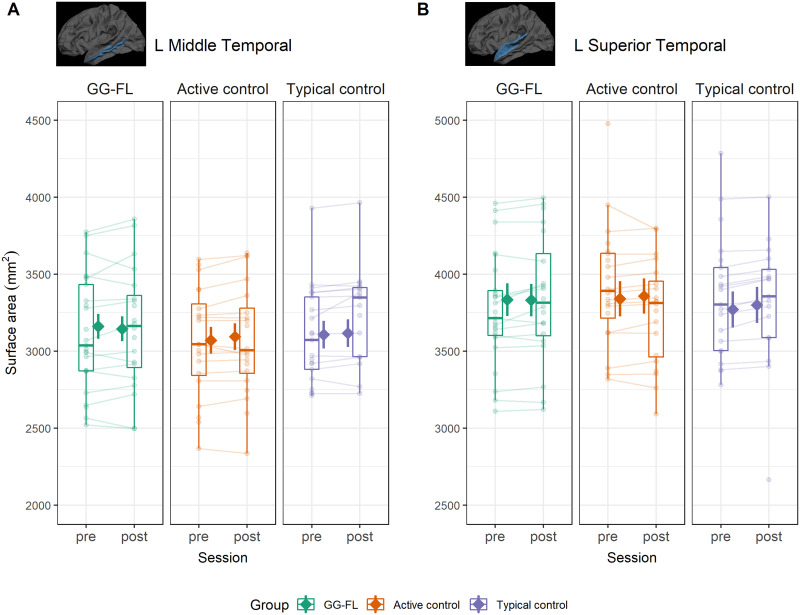
Group-specific longitudinal changes in surface area across sessions. Boxplots and semitransparent dots and lines represent individual subject longitudinal changes (raw data). The solid diamonds represent model-predicted area values with 95% confidence bands across the two time points for the three groups. Note that these estimates are derived from robust mixed-effects models, giving less weight to outlying observations. (A) Results for the left middle temporal gyrus. (B) shows the results for the left superior temporal gyrus.

Longitudinal analyses of SA did not reveal any main effects of session. A group-by-session interaction effect was present in the left middle temporal region, such that the AC group exhibited an increase in SA over time (ΔSA = 23 mm^2^, 95% asymptotic CI [4.18, 42.55], *z* = 2.39, *p* = 0.017, *p*_FDR_ = 0.051), but not the GG-FL group (ΔSA = −16 mm^2^, 95% asymptotic CI [−35.4, 3.59], *z* = −1.6, *p* = 0.110, *p*_FDR_ = 0.165) or TC group (ΔSA = 9 mm^2^, 95% asymptotic CI [−10.71, 29.26], *z* = 0.91, *p* = 0.363, *p*_FDR_ = 0.363). A similar pattern was also observed in the left superior temporal region, where increased SA was found for the AC group (ΔSA = 18 mm^2^, 95% asymptotic CI [5.03, 30.25], *z* = 2.74, *p* = 0.006, *p*_FDR_ = 0.009) and the TC group (ΔSA = 30 mm^2^, 95% asymptotic CI [16.62, 43.06], *z* = 4.42, *p* < 0.001, *p*_FDR_ < 0.001), but not the GG-FL group (ΔSA = −3 mm^2^, 95% asymptotic CI [−16.03, 10.62], *z* = −0.4, *p* = 0.691, *p*_FDR_ = 0.691). These effects are visualized in Figure 5. No further evidence was found pointing to group-specific interaction effects.

Supplementary Table 1 and Table 2 (available at https://doi.org/10.1162/nol_a_00122) provide all the results for both cortical thickness and surface area analyses irrespective of significance, which enables a comparison of effect sizes and can serve as a reference for future studies.

## DISCUSSION

The present study investigated the impact of preventive intervention on gray matter structure in preliterate children with and without a cognitive risk for developing dyslexia. First, we used anatomical MRI to examine whether cognitive risk is associated with differences in cortical gray matter features such as thickness and surface area before the intervention. Our results revealed higher surface area of the right supramarginal region in at-risk children at baseline, when compared to children without a risk. Then, by means of a longitudinal design, we assessed changes in these cortical measures by comparing (1) at-risk children following a digital phonics-based training (i.e., GG-FL group), (2) at-risk children engaging with games not related to reading (i.e., AC group) and (3) typically developing children (i.e., TC group). We found evidence of increased cortical thickness in the left supramarginal region, which specifically occurred in the GG-FL group, but not in the other two groups. In addition, lower cortical thickness and higher surface area across sessions were observed in certain bilateral regions for the AC group, but not the other two groups.

### At-Risk Pre-Reading Differences

In line with previous MRI studies in pre-readers, we showed that children at risk for dyslexia exhibit structural differences compared to children without a risk, which precede the onset of reading instruction (Im et al., 2016; Raschle et al., 2011). Specifically, our analysis demonstrated a higher surface area of the right SMG in at-risk children relative to the TC group, which was confirmed in analyses relying on another brain aparcellation method and in whole brain-analyses (see Supplementary Material). Accordingly, previous research has demonstrated familial risk-related differences in surface area in young children such as smaller inferior and middle temporal gyri bilaterally (Beelen et al., 2019) or atypical asymmetry of the planum temporale area (Vanderauwera et al., 2018). There is evidence to support more prenatal (rather than postnatal) influences on development of surface area (Jha et al., 2019; Kapellou et al., 2006; Raznahan et al., 2012), which could imply that the differences reported in the literature are driven by genetic risk. However, the present results suggest that these early individual differences may also be found in the case of cognitive (behavioral) risk, thus hinting at more complex gene-environment interactions. This conclusion is also consistent with a previous finding of cognitive risk-related white matter differences in pre-readers, as indexed by lower fractional anisotropy (Economou et al., 2022).

Concerning the direction of risk-related differences, previous studies reported reduced cortical surface area, thickness and volume measures in the presence of familial risk in pre-readers, mainly in the left hemisphere but also in certain right-hemispheric regions (Beelen et al., 2019; Black et al., 2012; Raschle et al., 2011). In contrast, our findings revealed a different pattern, showing that at-risk children have a higher surface area in a right-hemispheric region compared to children without. Anatomy of right-hemispheric structures has already been hypothesized to play a role in facilitating protective or compensatory mechanisms among at-risk children who develop typical reading skills (Yu et al., 2018). For instance, although less evidence exists on gray matter morphology, our results fit well with a study showing that white matter organization of right posterior fronto-parietal white matter was predictive of later reading outcomes among at-risk kindergarteners (Zuk et al., 2021). At the same time, studies in pre-readers who subsequently develop dyslexia have reported evidence more suggestive of compensatory (than protective) role of right hemisphere regions, such as a smaller right fusiform gyrus or altered developmental trajectories in the right hemisphere (Beelen et al., 2019; Phan et al., 2021). Thus, past and current findings support the idea that right hemisphere anatomy may support, but also constrain early literacy acquisition in at-risk children, and future research focusing on early neurodevelopmental trajectories is necessary to discern protective from experience-driven factors.

### Intervention-Related Differences in Thickness

Following 12 weeks of tablet-based training at home, we found evidence of cortical thickening in the left SMG, which was present in the at-risk GG-FL group, but neither the at-risk AC group nor the TC group. The specificity of this result to the GG-FL group and not the AC condition, suggests that the observed cortical change in this region is uniquely related to receiving early literacy training. The location of this intervention-related increase was also confirmed when whole-brain analyses or another brain parcellation method were used, showing the robustness of this effect to the analysis approach (see Supplementary Material). Past research showed that the left SMG is activated during language tasks requiring phonological processing (for a review see Price, 2012), which led to the proposition that this region constitutes part of the dorsal indirect route of reading, which involves the use of grapheme–phoneme mapping to successfully decode words (Jobard et al., 2003). Evidence from adult studies showing left SMG engagement when forming novel grapheme–phoneme associations further corroborates this proposition (Kaestner et al., 2022; Xu et al., 2020). A major component of the GG-FL training provided in the current study was the introduction and systematic training of auditory discrimination of phonemes and visual discrimination of graphemes, as well as establishing the associations between the two. Therefore, the assumed role of the left SMG in audiovisual integration and by extension reading, could provide a mechanistic explanation for the cortical plasticity we observed in this region after training grapheme–phoneme coupling.

Our observation of intervention-related thickness growth in the left SMG is in agreement with recent work by Romeo et al. (2018), which examined structural brain changes following intensive reading intervention in 6- to 9-year-old children with reading disability. Romeo and colleagues reported significant cortical thickening across the intervention period in several bilateral regions, including the left SMG. These changes were exclusively observed when intervention responders were compared to nonresponders. In addition, our findings fit well with recent evidence on cortical changes during the first years of reading development. Phan et al. (2021) reported continued gray matter maturation from kindergarten to second grade, which was observed in certain left-hemispheric regions including the left supramarginal and temporal cortex. Taken together, these findings support the idea that early reading development is a sensitive period for experience-driven changes in key regions of the reading network.

Two other studies have investigated gray matter plasticity in response to reading intervention in school-aged children with dyslexia with mixed findings: One observed bilateral increases in gray matter volume after an 8-week intervention (Krafnick et al., 2011), and the other study reported no changes in thickness or volume following a 3-month intervention (Partanen et al., 2020). This divergence in findings might be explained not only by differences in analysis and experimental setup, but also by intervention-related factors such as the language transparency, the intensity and content of the training program, as well as the target group receiving the training. For instance, the study by Romeo et al. (2018) employed an intervention that was delivered to small groups of pupils by trained staff, accumulating more than 100 hours in 6 weeks. This intervention aimed to improve a broad range of skills, from orthographic and phonological processing to reading accuracy and reading comprehension. The same intensive program was also shown to lead to widespread white matter plasticity in another cohort (Huber et al., 2018). In contrast, GG-FL training provided in the context of the present study was envisioned and developed as a tool to support early literacy acquisition and provide the basic building blocks for word decoding, such as learning and applying grapheme–phoneme mapping rules (Glatz et al., 2022). This distinction between broad versus narrow scope of intervention content may explain why the present study finds a more localized intervention effect as opposed to the widespread plasticity reported in the studies by Huber et al. (2018) and Romeo et al. (2018). It is also worth noting that the current study examined the impact of intervention in pre-reading children, thus focusing on a narrow window in early childhood compared to previous studies. Given the diverse findings reported thus far in past and current studies, we can conclude that while there is some evidence linking literacy intervention to measurable changes in gray matter properties, the behavioral and neuroplasticity outcomes are likely to vary depending on substantial differences among studies in the type and frequency of training, as well as the intended aims and participants of the program.

Notably, no evidence was found in the present study to support cortical plasticity in vOT cortex. The functional specialization of left vOT cortex to print is often viewed as the anatomical signature of emergent literacy and thought to occur during the initial stages of literacy development (Dehaene & Dehaene-Lambertz, 2016). This specialization is further supported by functional MRI evidence demonstrating activation changes in vOT cortex during learning of grapheme–phoneme associations in pre-reading children (Brem et al., 2010; Pleisch et al., 2019), as well as neurophysiological evidence revealing the emergence of bilateral sensitivity to letter processing from the pre-reading to the early reading stage (Fraga-González et al., 2021). In the current study, structural changes in the vOT cortex were examined by means of assessing properties of the fusiform gyrus. We did observe a trend for increased pre- to post-thickness in the fusiform gyrus for GG-FL compared to a declining thickness trend in the AC group (*β* = 0.11), but this interaction effect was not statistically significant (*p* = 0.263). Note that complementary whole-brain analyses did not reveal an intervention effect in vOT either (see Supplementary Material). Thus, it seems that despite the demonstrated learning that occurred during the training (Vanden Bempt et al., 2021), these behavioral changes could not be clearly linked to vOT structural plasticity, at least not within the time course of investigation. Given previous evidence showing that response to intervention is directly linked to cortical plasticity (Romeo et al., 2018), it might be an interesting future direction to examine specific differences in vOT plasticity within the intervention group, as a function of behavioral gains, for example, between intervention responders and nonresponders.

Besides the observed effects in the left SMG, a different group-specific pattern was observed in regional thickness development of the AC group (i.e., at-risk children engaging with games not related to reading). Specifically, this group exhibited significant thinning across sessions in the left superior temporal gyrus (*β* = 0.18) and right inferior temporal gyrus (*β* = 0.21), which was not present in the GG-FL and TC groups. Longitudinal data from children aged 5–11 years demonstrated bilateral reductions in cortical thickness, presumably due to the co-occurring increases in myelination during this period (Sowell et al., 2004). At the same time, Sowell et al. (2004) described significant thickening in bilateral perisylvian cortex and anterior inferior frontal cortex. Although the current study investigated a much narrower window in development, our observation of reduced cortical thickness, at least in the left STG for the AC group, might suggest differential development for at-risk children relative to typical development and/or at-risk children receiving preventive intervention. Given that the AC group did engage with tablet-based training for the duration of the intervention and does not represent the natural developmental trajectory of at-risk children (Oei & Patterson, 2013), this reduction in thickness of the AC group could also reflect an effect specifically linked to playing the control games. Yet, since children could choose between different games during the play session, it seems unlikely that a specific cognitive skill (or a set thereof) was systematically trained by such a diverse training, which would in turn lead to localized cortical changes. Ultimately, it is not possible as part of the present study to attribute the changes in the AC group as effects of tablet-based gaming, development effects or findings related to the limitations of longitudinal imaging.

### Intervention-Related Differences in Surface Area

Next to thickness, the present study also reports on longitudinal analyses in surface area. Although we did not find any evidence for GG-FL training-related changes in this measure, our analysis yielded a rather unexpected pattern of results, such that surface area increased across sessions for the AC group in the left middle temporal and superior temporal gyri. This increase was also present in the TC group, but only in the left superior temporal region, whereas no significant changes in area were observed in the GG-FL group. Although earlier hypotheses suggested that cortical thickness undergoes more dynamic changes compared to surface area at this age, our results are in line with the developmental trajectory of surface area. Previous research showed that surface area increases over time within the first two years of life (Jha et al., 2019; Lyall et al., 2015). Evidence on development of this measure throughout early childhood is very scarce, presumably due to the challenges of acquiring longitudinal MRI data at that age. Nevertheless, available studies estimate that surface area peaks between 8 and 13 years old depending on the region, thus supporting the conclusion of continued increase in childhood before decreases in adolescence (Tamnes et al., 2017; Wierenga et al., 2014). We note, however, that this pattern of regional surface area increase does not emerge in the GG-FL group, which makes it difficult to interpret this as a maturational effect. Alternatively, the group-specific differences we observed in longitudinal area changes could be influenced by environmental or demographic factors not explored within our study design. Lastly, it should be noted that the surface area effects described here did not emerge in complementary whole-brain analyses (see Supplementary Material), which suggests that these findings might not be robust and should be replicated in a larger sample.

### Underlying Mechanisms

Several underlying cellular processes, including changes in neuronal size and glial cell density, as well as vasculature, could influence the observed literacy-intervention-related changes in cortical thickness identified by MRI (Zatorre et al., 2012). One aspect that is suggested to differentiate between glial and neuronal responses to learning and experience is the timescale during which these events occur. For instance, evidence from rodent studies suggests a differentiation between transient glial changes versus persistent synaptic changes (Kleim et al., 2007; Yang et al., 2009). In humans, juggling training led to increased gray matter volume shortly after learning, which declined 3 months post-training (Draganski et al., 2004), thus consistent with the hypothesis of transient glial responses. On the other hand, maintenance of post-training gray matter changes has also been demonstrated (Draganski et al., 2006; Krafnick et al., 2011), which would be more in agreement with the persistent changes induced by synaptic plasticity.

Concerning the influence of other underlying neural changes, it is generally known that white matter is a contributing factor to changes in T1-related measures, which in turn may influence the estimation of metrics such as apparent gray matter volume (Tymofiyeva & Gaschler, 2021; Zatorre et al., 2012). However, it is less clear how white matter changes might influence changes in T1-derived CT. One study has probed this relationship across early childhood (1–6 yr), suggesting that CT and adjacent white matter myelin measures are largely independent measures, at least within this age range (Croteau-Chonka et al., 2016). The implication of these observations for the current work is that the intervention-related changes we reported on CT are likely to be independent of potential changes in white matter occurring during this period, at least changes specifically related to myelination. Given the rather mixed findings and the very limited evidence on learning-induced gray matter changes in young children, it is difficult to comment on which candidate processes may be contributing to the thickness changes observed in the current study. Future work should focus on examining both short- and long-term trajectories of gray matter plasticity across a wider age range in order to gain more clarity on the underlying mechanisms of learning plasticity.

### Limitations and Future Perspectives

A number of limitations are noted in the present study. One shortcoming is that this study did not explore brain-behavior associations in the context of intervention. If we aim to understand the underlying mechanisms of learning-related plasticity, it is important to establish a link between behavioral effects and experience-related brain changes (Zatorre et al., 2012). Despite a well-controlled longitudinal design and best efforts to develop a child-friendly imaging protocol, the sample size of the intervention group in the present study and particularly the number of complete pre–post datasets of sufficient quality was such that it prevented us from carrying out a proper analysis on how individual variability in post-intervention behavioral outcomes might relate to structural plasticity. Relatedly, we recognize that the exclusion of datasets due to image quality concerns resulted in a reduction of statistical power, meaning we might not be able to detect possible effects.

A final consideration concerns the anatomical specificity of our findings. Our primary analysis focused on a preselected set of cortical regions derived from a gyral-based brain parcellation, which means we may have missed the opportunity to examine smaller and/or widespread effects, potentially outside the canonical reading network, or effects related to experiencing structured tablet-based interaction. To this end, we performed two complementary analyses (described in detail in the Supplementary Material). In a first step, we repeated the ROI analysis using a more fine-grained cortical parcellation (gyri and sulci; Destrieux et al., 2010), in order to confirm that results were not dependent on the specific labeling method. In a second step, we examined both baseline and intervention-related effects using a whole-brain approach.

Overall, the two complementary analyses support the main conclusions of the current study. The finding of increased surface area in at-risk children at baseline emerged in all analyses with anatomical consistency, with only slight variations with respect to the precise location. Specifically, this effect seemed to be more extensive than just the SMG, covering a larger area of inferior parietal and superior temporal cortex. Likewise, the finding of literacy intervention-related increase in left perisylvian cortical thickness emerged in both complementary analyses, with similar indications that it extends beyond what was observed in the ROI analysis. Hence, the main findings of the study seem to be robust to the analysis approach. At the same time, there was no full correspondence between the ROI and whole-brain analyses (see Supplementary Material), particularly concerning the longitudinal effects seen in surface area. Keeping in mind that the longitudinal whole-brain analysis was performed on an even smaller sample (using only complete pre–post datasets), we suggest caution when drawing conclusions from these findings and encourage their future replication with a larger sample.

## CONCLUSION

In conclusion, this study explored the impact of preventive game-based training in preliterate children with an increased cognitive risk for developing dyslexia, during the optimal window for providing intervention. Our analyses yielded three main findings: 1) Behavioral risk for dyslexia is associated with structural brain differences in the right hemisphere even before the formal onset of reading acquisition, 2) engaging with a phonics-based training at home for 12 weeks leads to regional cortical thickening of the left SMG, and 3) developmental trajectories of cortical thickness and surface area may differ between at-risk and typically developing children; however, further research is needed to specifically investigate these longitudinal changes. Overall, the study contributes to the growing efforts in understanding the emergence of dyslexia and the underlying neurocognitive mechanisms supporting the remediation of reading difficulties.

## ACKNOWLEDGMENTS

We would like to thank all colleagues and master students for their assistance with MRI protocol preparation and data collection. We are grateful to Ron Peeters for technical MRI support. We are most thankful to all the participating families and children without whom this research would not be possible.

## FUNDING INFORMATION

Maaike Vandermosten, Onderzoeksraad, KU Leuven (https://dx.doi.org/10.13039/501100004497), Award ID: C14/17/046. Jolijn Vanderauwera, Fonds Wetenschappelijk Onderzoek (https://dx.doi.org/10.13039/501100003130), Award ID: 12T4818N.

## AUTHOR CONTRIBUTIONS

**Maria Economou**: Conceptualization; Data curation; Formal analysis; Investigation; Methodology; Writing – original draft. **Femke Vanden Bempt**: Investigation; Writing – review and editing. **Shauni Van Herck**: Investigation; Writing – review and editing. **Toivo Glatz**: Formal analysis; Writing – review and editing. **Jan Wouters**: Funding acquisition; Resources; Supervision; Writing – review and editing. **Pol Ghesquiere**: Funding acquisition; Resources; Supervision; Writing – review & editing. **Jolijn Vanderauwera**: Conceptualization; Supervision; Writing – review and editing. **Maaike Vandermosten**: Conceptualization; Funding acquisition; Resources; Supervision; Writing – reviewing and editing.

## DATA AVAILABILITY STATEMENT

Anonymized data and code to reproduce the figures of this study are publicly available at https://github.com/meconomou/Economou_etal_GMplasticity.

## Supplementary Material



## TECHNICAL TERMS

Cortical thickness: A measure of the thickness of gray matter of the human cortex.
Surface area: A measure of the surface area of gray matter of the human cortex.
Active control: A comparison group receiving training that lacks the main component of the target intervention (e.g., in this case literacy skills).
Grapheme-phoneme mapping: The knowledge that phonemes (individual sounds) correspond to graphemes (letters), which is key for learning to read.
