# Supplementary material for: Cortical Structure in Pre-Readers at Cognitive Risk for Dyslexia: Baseline Differences and Response to Intervention
Source: Neurobiol Lang (Camb). 2024 Jun 3;5(2):264–87. doi: 10.1162/nol_a_00122 (PMC11093402; doi:10.1162/nol_a_00122)
Supplement: Supplementary file 1 [file nol-5-2-264-s001.pdf]

Supporting Information

Supplementary Table 1. Post - pre change in cortical thickness per region and group, based on robust model-derived estimated marginal means. False discovery rate correction was applied per region.

Region	Group	Post - pre CT change (mm)	z ratio	95% asymptotic CI	p	p_{fdr}
L Fusiform	GG-FL	0.026	1.042	-0.023, 0.074	.298	.446
	Active Control	-0.014	-0.551	-0.064, 0.036	.581	.581
	Typical Control	-0.046	-1.730	-0.098, 0.006	.084	.251
L Inferior Temporal	GG-FL	0.012	0.419	-0.043, 0.067	.675	.675
	Active Control	-0.042	-1.435	-0.099, 0.015	.151	.257
	Typical Control	-0.041	-1.367	-0.1, 0.018	.172	.257
L Middle Temporal	GG-FL	0.020	0.860	-0.026, 0.067	.390	.390
	Active Control	-0.045	-1.823	-0.093, 0.003	.068	.205
	Typical Control	-0.028	-1.096	-0.078, 0.022	.273	.390
L Pars Opercularis	GG-FL	0.023	0.729	-0.038, 0.084	.466	.699
	Active Control	-0.061	-1.870	-0.124, 0.003	.061	.184
	Typical Control	0.005	0.158	-0.06, 0.071	.875	.875
L Pars Triangularis	GG-FL	-0.001	-0.026	-0.053, 0.052	.979	.979
	Active Control	-0.044	-1.595	-0.098, 0.01	.111	.332
	Typical Control	-0.022	-0.764	-0.078, 0.034	.445	.668
L Superior Temporal	GG-FL	0.015	0.729	-0.025, 0.054	.466	.546
	Active Control	-0.065	-3.136	-0.106, -0.024	.002	.005
	Typical Control	-0.013	-0.603	-0.055, 0.029	.546	.546

Region	Group	Post - pre CT change (mm)	z ratio	95% asymptotic CI	p	p_{fdr}
L Supramarginal	GG-FL	0.048	2.260	0.006, 0.09	.024	.071
	Active Control	-0.028	-1.252	-0.071, 0.016	.211	.316
	Typical Control	-0.017	-0.733	-0.062, 0.028	.463	.463
R Fusiform	GG-FL	0.025	0.982	-0.025, 0.076	.326	.583
	Active Control	-0.018	-0.675	-0.07, 0.034	.500	.583
	Typical Control	-0.015	-0.548	-0.069, 0.039	.583	.583
R Inferior Temporal	GG-FL	0.034	1.116	-0.026, 0.094	.265	.397
	Active Control	-0.068	-2.124	-0.13, -0.005	.034	.101
	Typical Control	-0.019	-0.585	-0.084, 0.045	.558	.558
R Middle Temporal	GG-FL	0.033	1.255	-0.019, 0.085	.209	.315
	Active Control	-0.034	-1.254	-0.088, 0.019	.210	.315
	Typical Control	-0.028	-0.984	-0.083, 0.028	.325	.325
R Pars Opercularis	GG-FL	0.000	-0.003	-0.061, 0.061	.997	.997
	Active Control	-0.006	-0.199	-0.069, 0.057	.842	.997
	Typical Control	-0.056	-1.679	-0.121, 0.009	.093	.280
R Pars Triangularis	GG-FL	-0.037	-1.673	-0.08, 0.006	.094	.141
	Active Control	-0.027	-1.173	-0.071, 0.018	.241	.241
	Typical Control	-0.060	-2.533	-0.106, -0.013	.011	.034
R Superior Temporal	GG-FL	0.013	0.478	-0.039, 0.065	.633	.870
	Active Control	-0.013	-0.475	-0.067, 0.041	.635	.870
	Typical Control	-0.005	-0.164	-0.061, 0.051	.870	.870
R Supramarginal	GG-FL	0.008	0.379	-0.035, 0.052	.705	.868
	Active Control	-0.019	-0.816	-0.064, 0.026	.414	.868
	Typical Control	-0.004	-0.166	-0.051, 0.043	.868	.868

Supplementary Table 2. Post - pre change in surface area per region and group, based on robust model-derived estimated marginal means. False discovery rate correction was applied per region.

**Region**	**Group**	**Post - pre SA change (mm^2**	**z ratio**	**95% asymptotic CI**	p	p_{fdr}
L Fusiform	GG-FL	11.93	1.09	-9.5, 33.37	.275	.800
	Active Control	6.22	0.58	-14.83, 27.27	.562	.800
	Typical Control	2.84	0.25	-19.1, 24.78	.800	.800
L Inferior Temporal	GG-FL	-6.98	-0.54	-32.12, 18.16	.586	.879
	Active Control	0.75	0.06	-23.67, 25.18	.952	.952
	Typical Control	-8.61	-0.66	-34.11, 16.88	.508	.879
L Middle Temporal	GG-FL	-15.91	-1.60	-35.4, 3.59	.110	.165
	Active Control	23.36	2.39	4.18, 42.55	.017	.051
	Typical Control	9.28	0.91	-10.71, 29.26	.363	.363
L Pars Opercularis	GG-FL	5.99	0.85	-7.81, 19.8	.395	.729
	Active Control	2.39	0.35	-11.13, 15.91	.729	.729
	Typical Control	4.08	0.57	-10.02, 18.18	.570	.729
L Pars Triangularis	GG-FL	8.05	1.23	-4.77, 20.88	.218	.427
	Active Control	2.94	0.46	-9.66, 15.54	.648	.648
	Typical Control	7.17	1.07	-5.96, 20.3	.285	.427
L Superior Temporal	GG-FL	-2.70	-0.40	-16.03, 10.62	.691	.691
	Active Control	17.64	2.74	5.03, 30.25	.006	.009
	Typical Control	29.84	4.42	16.62, 43.06	< .001	< .001
L Supramarginal	GG-FL	12.34	0.88	-15.17, 39.86	.379	.899
	Active Control	-1.71	-0.13	-28.14, 24.72	.899	.899
	Typical Control	-4.37	-0.31	-32.01, 23.27	.757	.899
R Fusiform	GG-FL	8.17	0.72	-13.97, 30.32	.469	.704

Region	Group	Post - pre SA change (mm^2	z ratio	95% asymptotic CI	*p*	p_{fdr}
	Active Control	16.80	1.50	-5.2, 38.81	.134	.403
	Typical Control	4.10	0.35	-18.74, 26.94	.725	.725
R Inferior Temporal	GG-FL	-4.16	-0.32	-29.69, 21.36	.749	.749
	Active Control	-12.25	-0.96	-37.19, 12.7	.336	.517
	Typical Control	-12.51	-0.95	-38.45, 13.42	.344	.517
R Middle Temporal	GG-FL	13.51	1.17	-9.22, 36.24	.244	.732
	Active Control	-3.16	-0.27	-25.77, 19.46	.784	.784
	Typical Control	7.54	0.63	-15.93, 31.01	.529	.784
R Pars Opercularis	GG-FL	0.86	0.14	-11.27, 12.98	.890	.934
	Active Control	0.51	0.08	-11.44, 12.45	.934	.934
	Typical Control	2.83	0.45	-9.58, 15.24	.655	.934
R Pars Triangularis	GG-FL	-4.30	-0.56	-19.21, 10.62	.572	.636
	Active Control	3.54	0.47	-11.11, 18.19	.636	.636
	Typical Control	14.80	1.90	-0.43, 30.02	.057	.171
R Superior Temporal	GG-FL	1.21	0.13	-16.73, 19.15	.895	.895
	Active Control	-3.35	-0.37	-21.08, 14.37	.711	.895
	Typical Control	-3.39	-0.36	-21.8, 15.02	.718	.895
R Supramarginal	GG-FL	3.11	0.31	-16.72, 22.94	.758	.758
	Active Control	-10.76	-1.11	-29.79, 8.27	.268	.739
	Typical Control	-6.94	-0.69	-26.76, 12.88	.493	.739

Confirmatory analysis using parcellations from Destrieux atlas

Parcellations of the Destrieux atlas implemented in Freesurfer (Destrieux et al., 2010) were used to confirm the effects described in the main text, which were based on the Desikan-Killiany

parcellation atlas (Desikan et al., 2006). Compared to the Desikan-Killiany (gyral-based atlas), the Destrieux atlas subdivides the cortex in sulcal and gyral subdivisions.

The results of the baseline analysis confirm the observed effect of larger surface area of the right perisylvian cortex in at-risk children compared to typically developing children (β = 0.46, 95% CI [0.09, 0.82], p_{boot} = 0.012). The effect was found in the right supramarginal ROI using the Desikan-Killiany parcellation but in the right superior temporal sulcus ROI using the Destrieux parcellation, which suggests the precise location is between the superior temporal gyrus and supramarginal gyrus.

Similarly, the results of the longitudinal analysis confirmed the presence of the literacy-specific intervention effect in the left supramarginal gyrus with the use of the Destrieux atlas. Specifically, we confirmed the observation of increased thickening over time in the GG-FL group (ΔCT = 58 μm, SE = 0.02, 95% asymptotic CI [0.01, 0.1], z = 2.51, p_{fdr} = 0.036), but not in the other two groups (AC: ΔCT = 5 μm, 95% asymptotic CI [-0.04, 0.05], z = 0.2, p_{fdr} = 0.843; TC: ΔCT = -10 μm, 95% asymptotic CI [-0.06, 0.04], z = -0.42, p_{fdr} = 0.843).

Finally, changes previously observed specific to the active control group were also confirmed when using the Destrieux parcellations. This applies to (1) the cortical thickness decrease found in the left superior temporal gyrus (z = -2.83, p_{fdr} = 0.014) and the right inferior temporal gyrus (z = -2.33, p_{fdr} = 0.060), and (2) the increase in surface area found in the left middle temporal gyrus (z = 2.94, p_{fdr} = 0.010).

Surface-based whole-brain analysis

Methodology

The ROI analyses presented in the main text were complemented by a whole-brain vertex-wise analysis, which was conducted using Freesurfer tools (mri_glmfit). All T1 images from both time points derived from the longitudinal processing stream were resampled to a standard brain (fsaverage) and smoothed with a 10-mm full-width half-maximum kernel.

To examine risk-related differences at pre-test, general linear models were constructed to test the whole brain for group differences in cortical thickness and surface area between at-risk children and typically developing children. For longitudinal data, symmetrized percent change (SPC) was defined as the dependent variable. SPC is the rate of change at each surface vertex with respect to the average cortical measure (thickness or area) across both time points and is more robust than a simple percent change, which refers to change only in terms of the first time point. To examine intervention-related effects, SPC was compared between groups (GG-FL, AC, TC) in a pairwise fashion. Note that to estimate SPC, data from both pre-test and post-test were required, therefore this analysis was only conducted using participants with a complete pre-post dataset (n = 16 for the GG-FL group, n = 15 for the AC group, n = 14 for the TC group).

In line with the methodology described in the main text, separate models were conducted for each metric (thickness, area) and hemisphere (left, right). All models were controlled for sex and surface area models were controlled for total hemispheric surface area. The whole-brain analyses were cluster-corrected for multiple comparisons using a Monte Carlo simulation (10 000 iterations) with a cluster-forming threshold of $p<.05$, cluster-wise $p<.05$ and Bonferroni correction for the two hemispheres. These thresholds were chosen to resemble the approach described in Romeo et al. (2018), so as to enable broader comparison with the only available

study examining whole-brain changes in cortical measures following reading intervention in young children.

Results

At baseline, no risk-related differences were observed in whole-brain cortical thickness. The only finding that emerged from the baseline analysis was that at-risk children had higher surface area compared to typically developing children, in a right hemisphere cluster including superior temporal, supramarginal and inferior parietal cortex (Supplementary Figure 1 and Supplementary Table 3). The peak of this cluster was located in the superior temporal gyrus and extended to other inferior parietal regions, which is anatomically consistent with the ROI analysis.

In terms of intervention effects, there were no differences in thickness changes between the GG-FL and AC group, however there were differences between the GG-FL and TC group in both hemispheres. Specifically, children in the GG-FL group showed increased cortical thickening in a left hemisphere cluster spanning superior temporal and inferior parietal cortex and a right hemisphere cluster spanning superior parietal and precentral/paracentral regions (Supplementary Figure 2 and Supplementary Table 4). We additionally observed increased cortical thickening in the AC group compared to the TC group, present in a right hemisphere cluster spanning inferior and middle frontal regions (Supplementary Figure 3 and Supplementary Table 5).

No effects were seen suggesting changes in surface area at the whole-brain level.

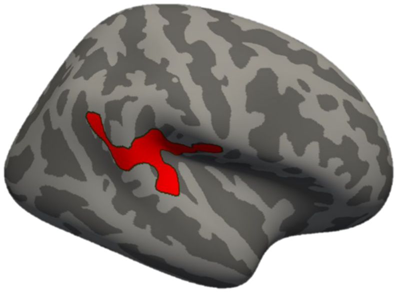

Supplementary Figure 1: Location of the cluster where surface area was higher in at-risk children compared to typically developing children following a whole-brain analysis. The cluster is shown on the inflated average template brain.

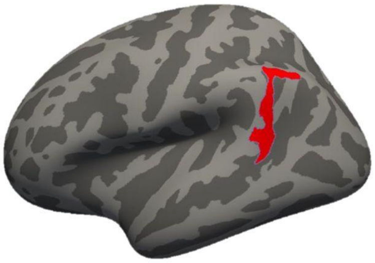

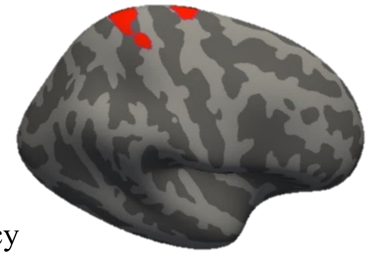

Supplementary Figure 2: Location of the clusters where symmetrized percent change in cortical thickness was higher in the literacy intervention group compared to the typical control group. The clusters are shown on the inflated average template brain.

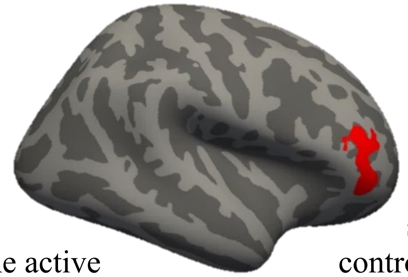

Supplementary Figure 3: Location of the cluster where symmetrized percent change in cortical thickness was higher in the active control group compared to the typical control group. The cluster is shown on the inflated average template brain.

Supplementary Table 3. Results of the whole-brain analysis examining baseline risk-related effects in surface area.

**Cluster region at peak**	**Area (mm^2)**	**Peak MNI coordinates** x,y,z	**Cluster-wise *p***
Right superior temporal	1534.62	60.8, -35.3, 16.2	.00818

Supplementary Table 4. Results of the whole-brain analysis examining differences in symmetrized percent change in cortical thickness between the literacy intervention group and typical control group.

**Cluster region at peak**	**Area (mm^2)**	**Peak MNI coordinates** x,y,z	**Cluster-wise *p***
Left banks of the superior temporal sulcus	847.72	-50.9, -46.6, 10.7	.00180
Right superior parietal	1229.03	17.9, -43.3, 64.8	.00020

Supplementary Table 5. Results of the whole-brain analysis examining differences in symmetrized percent change in cortical thickness between the active control group and typical control group.

**Cluster region at peak**	**Area (mm^2)**	**Peak MNI coordinates** x,y,z	**Cluster-wise *p***
Right pars orbitalis	739.79	42.8, 38.2, -4.7	.01117
